# Nutrient Intake, Excretion and Use Efficiency of Grazing Lactating Herds on Commercial Dairy Farms

**DOI:** 10.3390/ani10030390

**Published:** 2020-02-28

**Authors:** Sharon R. Aarons, Cameron J. P. Gourley, J. Mark Powell

**Affiliations:** 1 Agriculture Victoria Research, Ellinbank Dairy Centre, Victorian Department of Jobs, Precincts and Regions, 1301 Hazeldean Road, Ellinbank, VIC 3821, Australia; Cameron.Gourley@agriculture.vic.gov.au; 2 US Dairy Forage Research Center, USDA Agricultural Research Service, 1925 Linden Drive West, University of Wisconsin, Madison, WI 53706, USA; jmpowel2@wisc.edu

**Keywords:** grazed pasture intake, supplementary feed, metabolizable energy, feed nutrient intake, macronutrients, animal nutrient use efficiency, manure, nitrogen, phosphorus, potassium

## Abstract

**Simple Summary:**

Excess nutrients on dairy farms can affect soil and animal health and have negative impacts on the environment. More nutrients are usually brought onto farms in animal feed than in fertilizer and, as dairy cows do not efficiently use feed nutrients to produce milk, most consumed nutrients are excreted in dung and urine. Estimating nutrients excreted by cows relies on measuring feed and nutrient intake. However, measuring pasture nutrients consumed by cows that graze on commercial farms is complicated. We modified the ‘Feeding Standards’ approach to estimate daily pasture dry matter and nutrient intake and nutrients excreted using data and samples readily available on commercial dairy farms. These data were collected on five visits in different seasons from 43 grazing system farms located in diverse climatic zones. Although these herds graze daily, the lactating cows only obtained slightly more than half their dry matter intake from pasture. Each day, on average, we estimated that a grazing cow excretes 433 g nitrogen, 61 g phosphorus, 341 g potassium, 44 g sulphur, 92 g calcium and 52 g magnesium on these farms. Using this approach to estimate nutrients excreted by grazing animals on dairy farms can assist farmers improve nutrient distribution and fertilizer requirements.

**Abstract:**

Estimating excreted nutrients is important for farm nutrient management, but seldom occurs on commercial grazing system farms due to difficulties in quantifying pasture intake. Nitrogen (N), phosphorus (P), potassium (K), sulphur (S), calcium (Ca) and magnesium (Mg) intake, excretion and use efficiency of 43 commercial dairy herds grazing pasture were calculated to understand the range in nutrient intake and excretion in these systems. Milk production, feed (grazed and supplement), as well as farm and herd management data were collected quarterly on representative farms located in temperate, arid, subtropical and tropical regions of Australia. Lactating herd sizes on these farms averaged 267 (30 to 1350) cows, with an average daily milk yield of 22 (9 to 36) kg/cow per day and the herds walked from <0.01 to 4 km/day on a variety of terrains. The mean total metabolizable energy (ME) required by cows in the herds was estimated to be 195 (116 to 289) MJ/cow per day. Although these farms are considered grazing systems, feeding strategies ranged from total dependence on pasture to total mixed rations (TMRTMR) and consisted of a wide variety of nutrient and energy contents. Mean pasture dry matter intake (DMI) (9 kg/cow per day, from 0.1 to 22 kg/cow per day) was just over half of total DMI. Dietary concentration of crude protein, P, K, S, Ca and Mg concentrations were, on average, 19%, 0.45%, 2.1%, 0.29%, 0.65%, and 0.3%, respectively, for all herds and, except for N, supplement nutrient concentrations were always more variable than pasture. Approximately 72% and 88% of diets provided greater than recommended P and N intakes, respectively. Calculated mean N, P, K, S, Ca and Mg excretions were 433, 61, 341, 44, 92 and 52 g/cow per day, respectively. Of the farm characteristics examined, residual maximum likelihood (REML) analysis indicated that daily excreted N, P and S were significantly related to per ha milk production, and excreted P, K and Mg were related to percentage of herd DMI provided as supplement. Mean use efficiencies by cows of N, P, K, S, Ca and Mg were 21%, 25%, 9%, 16%, 23% and 4%, respectively. These estimates of nutrient excretion and feed nutrient use efficiencies can be used to improve nutrient management on grazing system commercial dairy farms.

## 1. Introduction

Nutrient accumulation that occurs on many dairy farms worldwide can lead to environmental pollution, through gaseous emissions and nutrient losses to waterways [1]. In most dairy production systems, imported feed contributes a greater source of nutrients than fertilizer inputs [2,3,4], and even in grazed dairy systems, feed P and K were greater than fertilizer applications [5]. Substantial inputs of feed nutrients along with the low use efficiencies by dairy cows of ingested nutrients leads to greater proportions excreted in faeces and urine than exported in milk [6,7]. Consequently, accounting for excreted nutrients is needed to ensure nutrient inputs at the farm scale are appropriately managed for both environmental and production benefits. Quantifying excreted nutrients for improved management of manure occurs more routinely in confinement-based systems due to the relative ease in measuring dietary intakes, with this information used to meet environmental and production objectives [7]. The relative ease of measuring DM intakes in confinement-based dairy production systems has assisted with quantifying excreted nutrients for improved manure management [8], and to meet environmental and production objectives (e.g., [9,10]). By contrast, measuring pasture intake in grazing systems is more challenging due to the interactions of the grazing animal and feed sources (pasture and supplements) as influenced by farm management practices [11]. Techniques such as herbage estimation, internal or external markers, and calculation of net energy requirements have been compared [12,13,14]. Methods such as alkane markers are better suited for research purposes, while calculation of net energy requirements can be more easily used to estimate cow intakes on commercial farms. The Cornell Net Carbohydrate and Protein System [15], and the GrazeIn [16] models have been used to estimate pasture intake and milk yield, while the energy requirements to produce milk has been used in a ‘back-calculation’ approach [17] to estimate pasture intake. By using these or similar methods to calculate dietary intake, nutrient excretion on commercial dairy farms has been estimated. The methods range from more data-intensive sampling strategies [10,18,19], where data were collected on a small number of farms, to more rapid monitoring approaches, where data were collected from larger numbers of farms [20]. Typically, however, pasture-based grazing systems were not included in these studies, and where commercial farms with grazing herds were monitored, data were only collected from a few farms [21]. In this paper, a methodology for estimating pasture dry matter and nutrient intakes of grazing lactating herds that is based on metabolic requirements of the cows, and which uses data readily collected from commercial dairy farms, is used to calculate excreted nutrients and nutrient use efficiencies.

## 2. Materials and Methods

Forty-three commercial dairy farms from a variety of grazing management systems in all climatic zones of Australia were selected, from an initial pool of 124 dairy farms (Figure S1, see Gourley et al. (5) for the detailed methodology). Briefly, a stratified-random process, rather than a random sampling approach, was used to identify a diversity of farms. Six criteria used in farm selection were proportional representation in the major dairy regions nationally, per hectare milk production, farm area (ha), use of irrigation, soil P sorption and inclusion of organic farms. An iterative optimisation routine identified 44 farms, with one farm excluded due to insufficient data. The participating farmers were interviewed on five separate occasions (summer, autumn, winter and spring 2008 and summer 2009), with feed and milk samples collected on the farms after the interviews. Data and sample collection typically occurred over a relatively short period (2 to 3 h) at each interview.

### 2.1. Survey Data

Similar approaches combining collection of both farm management data and samples have previously been used (e.g., [20,22]) to assess nutrient flows on dairy farms. In this study project, farmers were always interviewed by the same trained technical staff, using a semi-structured questionnaire designed for recording data about the production and intake of lactating herds present on the farm at each interview. The farmers were asked about the amount of milk produced, cow numbers in the herds, their stage of lactation, the average age of animals, and an estimated average weight of cows in the lactating herd (Table S1). Diets provided to herds were described by the farmers, including the types and estimates of the amounts consumed as well as estimates of wastage (Table S2). Data from the survey (and feed and milk analyses) resulted in 227 herd records, as 6 farms milked two herds on at least one visit and one farm milked two herds at all times.

### 2.2. Dietary Intake

The ‘Feeding Standards’ approach [17] for quantifying annual pasture dry matter intake (DMI) was modified to calculate DMI at each interview date, based on the estimated metabolizable energy (ME) requirements of the lactating herds,

where

ME_PastureIntake_ (MJ/day) = ME_TotalReq_ (MJ/day) – ME_Supplementfed_ (MJ/day) (1)

and

ME_TotalReq_ = ME_Maintenance_ + ME_Graze_ + ME_Pregnancy_ + ME_MilkProduced_ + ME_Activity_ (2)

The equations for each calculation were as follows:

ME_Maintenance_ (MJ/day) = 1.4 × 0.28 × ([LiveWt]^0.75^) × Exp (−0.03 × 2.5) ÷ [k_m_] × [NLactn1] + 1.4 × 0.28 × ([LiveWt]^0.75^) × Exp (−0.03 × 4.5) ÷ [k_m_] × [NLactn2+] (3)

ME_Grazing_ (MJ/day) = 0.1 × [ME_Maintenance_] (4)

ME_Pregnancy_ (MJ/day) = [NMidLactn] × 349.16 × 0.0000576 × Exp(−0.0000576 × 69) × Exp(349.22 − 349.16 × Exp(-0.0000576 × 69)) ÷ [k_c_] + [NLateLactn] × 349.16 × 0.0000576 × Exp(−0.0000576 × 170) × Exp(349.22 − 349.16 × Exp(−0.0000576 × 170)) ÷ [k_c_] (5)

ME_MilkProduced_ (MJ/day) = [NAnimals] × [MilkPerCow] × ((0.0381 × [Fat]) + (0.0245 × [Protein]) + (0.0165 × [Lactose])) ÷ [k_l_] × 1.1 (6)

ME_Activity_ (MJ/day) = ((0.0026 + (0.028 × [Vert])) × [WalkDist] × [LiveWt] ÷ [k_m_] × [NAnimals]) × [MilkingTimes] × 2 (7)

where

(i) [NLactn1] and [NLactn^2+^] are the number of cows in their first and in their second or greater lactations, respectively;
(ii) [LiveWt] is the estimated liveweight of the cows and for the purposes of estimating daily energy requirements is assumed to be constant for the day of visit to the farm;
(iii) [NMidLactn] and [NLateLactn] are the number of cows in mid and late lactation respectively, where the lactation length was 305 days, divided evenly into early (102), mid (103 to 204) and late (205+) lactation;
(iv) [MilkPerCow] is milk produced (L) per cow taking into consideration the number of heifers [NLactn1] and older cows [NLactn^2+^];
(v) [WalkDist] is the distance walked (km) by the herd from the dairy shed to the middle of each paddock;
(vi) [Vert] is the steepness factor according to Heard et al. [17], based on the representative terrain (flat, undulating, steep) at each farm;
(vii) k_m_: 0.02 × [ME_Diet_] + 0.5;
(viii) k_c_: 0.133;
(ix) k_l_: 0.02 × [ME_Diet_] + 0.4;
(x) ME_Diet_: 10 MJ/cow per day (Heard pers comm).

The ME consumed in supplements (ME_Supplementfed_) was summed for all supplements fed to the lactating herd, accounting for estimates of wastage, and based on the ME concentration of all supplements fed.

ME_Supplementfed_ (MJ/day) = DMI_Supplement_ × ME Conc_Supplement_ (8)

Pasture DMI was calculated as

DMI_Pasture_ = ME_PastureIntake_ ÷ ME Conc_Pasture_ (9)

using the ME_PastureIntake_ as estimated in equation [1] and the ME concentration of the pasture samples collected for each herd on each farm at each interview.

### 2.3. Nutrient Use Efficiency

Using the calculated pasture DMI, nutrients excreted (g/cow per day) were obtained by subtracting total dietary nutrient intake from milk nutrients secreted. Animal nutrient use efficiencies were calculated as the proportion of nutrients secreted in milk to those consumed.

Animal nutrient use efficiency (%) = Milk nutrient ÷ Nutrient Intake × 100(10)

### 2.4. Sample Collection and Analysis

Feed and milk samples were collected on all farms after each interview for subsequent chemical analysis. Approximately 400 mL of milk (from all milkings for the day) was collected after thorough agitation of the vat (bulk tank), placed on ice, returned promptly to the laboratory and frozen (−20 °C) until chemical analysis. Separate grab samples of all dietary supplementary feeds (e.g., grains and mixed concentrates, by-products, feed additives such as minerals, mixed rations, silage, hay) were collected, totaling 830 samples. The exact weights of non-forage supplements provided by farmers to herds in the dairy shed were confirmed by weighing (in triplicate) the amounts dispensed by feeders. Where necessary the amounts stated by farmers were corrected to reflect the actual amounts dispensed to the animals. A minimum of 20 grab samples of pasture (to post-grazing height) were collected and composited from pastures representative of paddocks recently grazed by the lactating herd on each farm.

Supplementary feeds and pasture samples were oven-dried (65 °C, 72 h) then ground to pass a 2 mm screen. Subsamples were dried at 100 °C for 24 h for calculating percent DM. Feed and milk samples were analysed by George Weston Technologies, Sydney, currently operating as Weston Food Laboratories. Crude protein (CP) in feed and milk was measured according to AOAC methods (CP ÷ 6.25 was used to calculate total N concentration in feed; CP ÷ 6.38 was used to calculate total N concentration in milk) [23]. Total P, K, S, Ca and Mg in feed were measured by inductively coupled plasma optical emission spectrometry after digestion in hydrochloric and nitric acid.

### 2.5. Data and Statistical Analysis

Data collected at each interview visit as well as sample analytical data were entered into an Access (Microsoft Office, Armonk, NY, USA) database and queries based on the equations above were used to calculate dietary and nutrient intakes, milk nutrient secretion, nutrient excretion and feed nutrient use efficiencies. Energy corrected milk was calculated according to Tyrrell and Reid. [24], where the density of milk was used to convert milk volume to kg.

ECM (kg) = (Milk yield (L) × 1.0295 × (376 × fat% + 209 × protein% + 948)) ÷ 3138(11)

For 15 out of 227 records where milk fat and protein concentration data were not available, average concentrations were calculated using either data from the other interview samples for a farm, if data from more than three interviews were available (3 farms), and for three other farms using the average of the whole dataset.

Statistical analyses were undertaken using Genstat 17 (VSN International, Wood Lane, Hemel Hempstead, UK) and RStudio© 0.99.891 (R version 3.2.3), and RStudio and S-plus 8.1 (TIBCO Spotfire, CA, USA) were used for graphical representation. Residual maximum likelihood (REML, Genstat) was used to analyse relationships between nutrient excretion and farm characteristics, such as herd size. The fixed model comprised an explanatory variate (e.g., herd size), or the explanatory variate crossed with interview date or season (e.g., herd size × season), and the random model consisted of the factor ‘farm’. In these analyses tests for fixed effects were determined by sequentially adding terms to the fixed model. Although interviews were conducted on five occasions on each farm, the herds at each interview consisted of different cows that were at different stages of lactation and which received different diets. Consequently, ‘interviews’ were not nested in ‘farm’ in the random model. Pearson correlation coefficient and linear regression analyses were performed in R using the cor and lm functions, respectively. Linear regression analyses investigated relationships between the explanatory variables total DMI or supplementary DMI and response variables ECM or FCE (feed conversion efficiency; kg ECM/kg DMI). For analyses of relationships at each interview visit, or different seasons, or for diets with low (L ≤ 20%) or high (H ≥ 20%) crude protein (CP), the data were subset (e.g., lm(FCE~DMI, subset = (CP.level = “L”))). Correlation coefficients and p-values were generated in a four column matrix using flattenSquareMatrix(cor.prob()). The regressions and correlations were represented graphically or tabulated. All data met assumptions of normality and constant variance based on residual graphs of histograms, fitted value and quantile plots.

## 3. Results

### 3.1. Dairy Farm Characteristics

The number of milking cows in each herd on these commercial farms for all interviews ranged from 30 to 1350, with approximately three times as many cows in their second or greater lactation as were in their first, and an estimated average age of the herds of 4 years old (Table 1). Most herds were Friesian (20 farms), or Jersey/Friesian crosses (approximately 16 farms), with four farms milking Aussie Red and Brown Swiss or Ayrshire cows and two farms milking Illawarra breeds. Across all interviews (227 records), most cows were in mid lactation (193 records), while for 25 records, all or the majority of the herd were in late lactation and for 19 records most or all animals were in early lactation. The herds produced, on average, 22 kg ECM/cow per day, although the yield of the highest producing cows was four times greater than the lowest. The distances the herds walked ranged from immediately adjacent to the dairy shed to over 4 km one way. All terrain types (flat, undulating, steep) were recorded in this study, and all paddocks on each farm were assigned the same terrain type. Similar numbers of the farms were classified as flat (20) and as undulating (22) with only one farm classified as steep.

Of the calculated daily total ME (Table 2), per cow maintenance requirement ranged from 46 to 66 MJ/cow per day when the estimated per herd maintenance ME was divided by the number of cows in each herd. Energy required for pregnancy was most variable (CV = 68%), as herds on seven farms had all cows in early lactation (i.e., less than 102 days in milk) at one of the interview dates and as such, were considered to be expending little energy in pregnancy at that time, while cows on the remaining farms were in mid to late lactation and expended a maximum of 4.78 MJ/cow per day. The calculated mean energy required for producing milk was approximately twice that for maintenance at 124.5 MJ/cow per day. The cows expended an average of 9 MJ/cow per day on walking, although this ranged from 3.7 to 24.1 MJ/cow per day.

### 3.2. Dietary Intake

Dairy farms in this study, while considered grazing-based systems, utilised various feeding strategies, from complete dependence on pasture to total mixed rations, with diets containing a wide range of nutrient and energy contents (Table 3). Cows grazed at least 11 different types of pasture and also grazed crops on some farms at some interview dates. Only herds on Farms 32, 33 and 34 at the last interview date were not provided pasture and therefore did not graze. Similarly, Farms 1 and 36 did not provide supplements to their herd on at least one occasion over the study. Mean ME of the different feeds was consistent across feed types, ranging between 8 and 13 MJ per day. However, by-products were most variable in ME, with analysis of some individual feed types reporting 0 MJ/day (e.g., vinegar, sea salt), while others were as high as 17 MJ/day (e.g., linseed meal). By-products also had very variable N contents with soybean meal having highest concentrations, although the highest mean N concentrations were observed for pasture. Forage crops had the highest mean K concentrations followed closely by pasture which also had the greatest range in K concentration. Mineral supplements contained the highest P, S, Ca and Mg concentrations and had the greatest range in values. For example, lime feed supplements provided in a temperate region farm had the maximum P and Ca concentrations, while a commercial mineral mix fed on a more arid region farm had the highest Mg concentration. Both of these feed types were not a constant feature of the herds’ diets over the year of this study only being supplied in small amounts and on three occasions on both farms.

Supplement N, P, K, S, Ca and Mg intakes for all interview dates and farms averaged (range) 226 (7–667), 42 (2–155), 124 (3–511), 21 (1–72), 64 (2–216), and 28 (1–88) g/cow per day, respectively (data not presented). On average, supplement nutrient intake on these farms was 42%, 50%, 36%, 42%, 53% and 51%, respectively, of total nutrient intakes and ranged from 0 to 100% for all nutrients.

Based on ME requirements of the cows and the total ME fed in supplements, calculated pasture DMI ranged from 0.04 to 22.5 kg DM/cow per day, excluding those animals that did not access pasture and therefore had no pasture intake (Table 4). Mean and ranges in pasture DMI were similar to those for supplement DMI and constituted an average of 51% (median 53%) of total DMI for all farms. Calculated pasture DM consumed showed a strong 1:1 relationship with farmer estimates of pasture intake (correction bias of 0.9935, (Figure S2), although Lin’s correlation coefficient (0.66) showed considerable deviance around the line of best fit resulting in a concordance of 0.65 (95th percentile confidence limits of 0.57 to 0.73).

**Figure 1:**
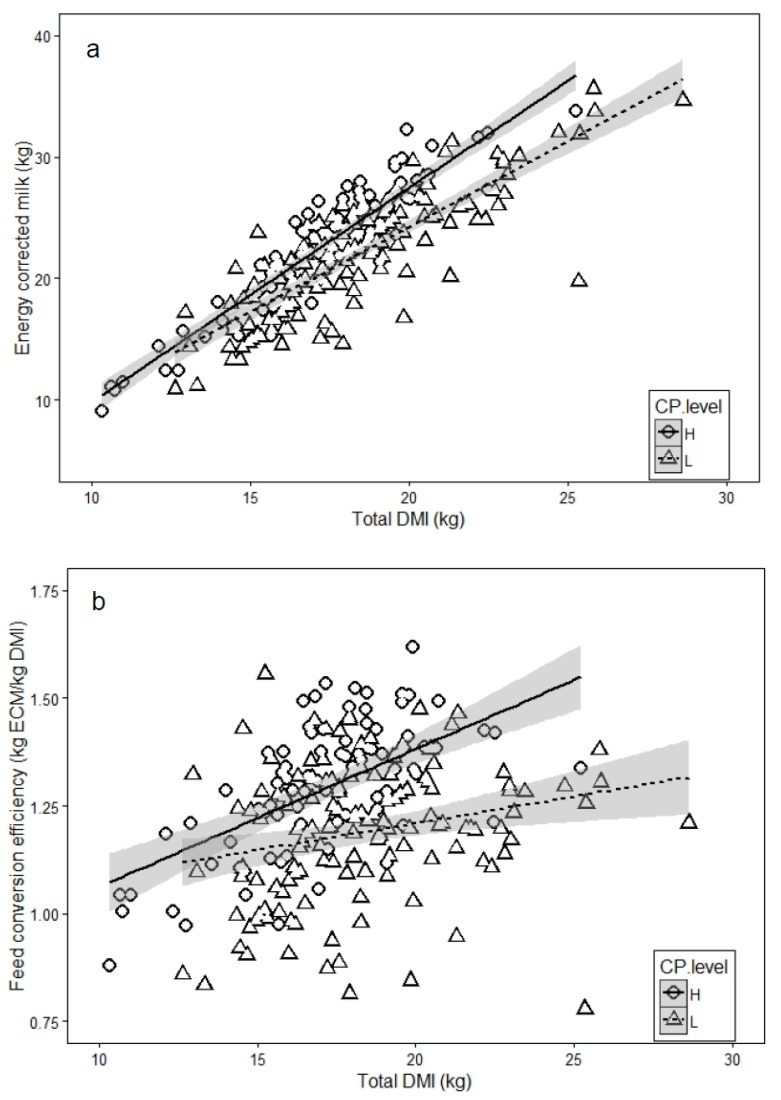
Relationship between energy corrected milk (ECM); (**a**) or feed conversion efficiency (**b**) and total dry matter intake (DMI) by lactating dairy herds on 43 grazing system dairy farms at low (L; <20%) and high (H; >20%) dietary crude protein (CP).

Lower CV (17%) in estimated total DMI (10 to 29 kg DMI/day) was observed for all farms and interviews (data not presented) compared to that for pasture (51%) and supplements (54%) DMI. Despite the variety of diets offered nationally, both milk yield (R^2^ = 0.77, data not presented) and ECM (R^2^ = 0.75) were significantly (*p* < 0.008) related to total DMI at all interviews (Table 5), although the response to supplement DM fed was not significant in spring. Greater increases in milk production in response to total DMI were observed for those diets that were above 20% CP in these systems (Figure 1). Feed conversion efficiencies ranged from 0.8 to 1.62 kg ECM.kg DMI^−1^ and showed a poor correlation to DMI, particularly to estimated pasture DMI (Table 6).

Graphs were prepared using R Studio 0.99.891. Shaded areas represent 95% confidence intervals around the regression lines.

### 3.3. Feed Nutrient Intake, Excretion and Animal Use Efficiency

Calculated nutrient intakes (g/cow per day) were highly variable with pasture N intake having the lowest CV and pasture Mg intake the highest (Table 4). Supplement N, P, K, S, Ca and Mg intakes (*n* = 227) averaged (range) 226 (7−667), 42 (2−155), 124 (3−511), 21 (1−72), 64 (2−216), and 28 (1−88) g/cow per day, respectively (data not presented). Supplement nutrient intakes varied between interview dates for all farms and no farms ever had both maximum and minimum supplement intakes. On average, supplement nutrient intake was 42%, 50%, 36%, 42%, 53% and 51%, respectively, of total nutrient intakes—ranging from 0 to 100% for all nutrients.

Pasture and supplement nutrient intakes were summed and divided by total DMI to give dietary CP, P, K, S, Ca and Mg concentrations, which, on average (range), were 19% (9−30), 0.45% (0.21−0.75), 2.1% (0.8−4.0), 0.29% (0.14−0.56), 0.65% (0.17−1.34), 0.3% (0.14−0.6), respectively. Except for N, supplement nutrient intake concentrations were always more variable than pasture nutrient intake concentrations. Maximum total N, K and S intakes were four times minimum intakes for these nutrients and six, eight and five times minimum dietary P, Ca and Mg (respectively, Table 7). Mean calculated N, P, K, S, Ca and Mg excretion was 433, 61, 341, 44, 92, and 52 g/cow per day respectively, and differences between the maximum and minimum were greater than for the calculated intakes. The CV was lowest for excreted N, similar for K and S and highest for Ca and the excreted data were not skewed or kurtotic. On average, close to 80, 75, 90, 85, 80 and 95% of N, P, K, S, Ca and Mg intakes were excreted by these herds.

Residual maximum likelihood analysis (Table 8) was undertaken to identify any relationships between estimated daily excretion of nutrients and farm characteristics such as annual milk production, farm size or supplementary feed importation. Where significant effects of farm characteristics on excreted nutrients were observed these were generally positive. Only N excretion (*p* = 0.013) was positively related to farm size, while N and S excretion were strongly (*p* < 0.004) positively related to herd size, stocking rate and total milk produced on the farm. Nitrogen, P and Mg excretion increased with per cow milk production, while only N, P and S excretion were related to per hectare milk produced. Both P and Mg excretion increased (*p* < 0.001) as the percent of dietary ME that was imported increased. An indication for P excretion (*p* = 0.057) to be related to percent of the farm area that received irrigation was observed. Negative relationships were observed between K and Ca excretion and farm characteristics. Potassium excretion was significantly related to importation of supplementary ME (*p* = 0.007), and Ca excretion was related to herd size (*p* = 0.027) and stocking rate (*p* = 0.046). Highly significant season effects were observed for relationships between Mg excretion and per cow milk production (*p* = 0.007) with weaker relationships observed for the other characteristics (0.013 < *p* < 0.046). Neither season nor interview date influenced relationships between K or Ca excretion and the farm characteristics tested, but significantly (*p* < 0.001) influenced relationships between the other nutrients excreted and farm characteristics (data not presented).

## 4. Discussion

Measurement of animal intakes on commercial dairy farms is critical for assessing within-farm nutrient flows and how the latter influence nutrient deposition and accumulation; as a means of identifying nutrient management points of intervention. Due to difficulties in measuring nutrient intake by grazing animals, a number of methods have been compared for measuring pasture DM intake [12,13,28], and while internal and external marker techniques are more suited to research, few approaches are ideal for use in commercial settings [29]. For instance, herbage disappearance methods, based on observation or use of methods such as the rising plate meter (RPM), were shown to be useful for measuring pasture intake by a herd. However, these methods are time-consuming and difficult to implement on an ad hoc basis on commercial dairy farms. Furthermore, while the RPM provides reasonable estimates of pasture removal, this method does not account for any pasture trampled or defecated on, or consumed on the way to or from the paddock [14]. The RPM also requires repeated calibration making it more difficult to deploy for rapid assessment of DMI on commercial farms. Methods based on animal performance provide estimates of animal intakes [12,13] that are at least as accurate as other more costly and time-consuming herbage methods [29]. The Cornell Net Carbohydrate and Protein System (CNCPS), has been used to evaluate effects of dietary changes on milk production, nutrient excretion and the economic impacts of these changes [15], and require inputs—many of which are not typically available on commercial dairy farms. Similarly, O’Neill et al. [16] developed regression equations to predict DMI from animal and plant production characteristics, but their models require pre and post grazing height measurements and should not be directly applied to systems other than Irish dairy farms. Heard et al. [17] evaluated the ‘Feeding Standards’ approach which simulates annual forage consumption on grazing dairy farms and considered it an improved method based on the high concordance to experimentally determined pasture DMI.

### 4.1. Modified ‘Feeding Standards’ Approach

The ‘Feeding Standards’ approach was modified in this study to allow for calculation of daily rather than annual pasture intakes by grazing herds and therefore to account for seasonal differences on commercial farms. Not surprisingly, milk yield across the study farms varied, as they were selected to be representative of the systems that occur in a variety of climatic zones and regions nationally [5]. The maximum reflected the high producing cows and systems with larger animals while the minimum was associated with a farm where the cows were in late lactation and their intake comprised only grazed pasture. The predominantly seasonal nature of milk production also contributed to the variability in milk yield across the year of the study.

Heard et al. [17] observed that of the livestock parameters tested in their sensitivity analysis, liveweight and terrain were the most important although supplemental feed inputs had generally larger impacts on estimation of forage consumption. In this study, the terrain the animals traversed was accounted for, although a single factor was applied to the whole farm. Animal liveweights were based on herd average estimates provided by farmers. For the range of herd weights in this study, daily ME requirements for maintenance could vary from 40 up to 70 MJ/day for Jersey and Friesian animals [30], with the estimates for this study (46 to 66 MJ/cow per day) falling within those ranges. We calculated daily ME maintenance requirements incorporating either a 10% increase or decrease in the herd average liveweights provided by the farmers, which resulted in a 7.4% increase and a 7.6% decrease, respectively, in estimated maintenance ME. Mean (minimum, maximum) estimated pasture DMI increased by 0.388 kg (0.301 kg, 0.628 kg) per cow per day or decreased by 0.397 kg (0.644 kg, 0.309 kg) per cow per day, which, like Heard et al. [17], was similarly less than a 5% change in DMI. While a number of other breeds were included in this study, maintenance requirement multipliers for most breeds only ranged from 0.07 to 0.074 [15], suggesting that the variation in breeds in this study may not have had a big impact on ME estimates and explaining the narrow range in calculated maintenance ME requirements.

Estimation of dietary ME is difficult to accommodate in studies on commercial dairy farms, with Reeves et al. [12] observing that accurate feed quality data minimizes errors in estimating intakes. The ME data for supplements and pasture in this study were based on chemical analysis of over 800 samples of different feeds provided over the year of the study and collected on farms [26]—many of which were not previously reported in feed databases. Thus, the feed sample data in this study enabled a more accurate calculation of DMI, reflecting seasonal variations in ME contents which would otherwise not have been possible with the use of book values. Assessments of wastage were also shown to be important in sensitivity analyses of the ‘Feeding Standards’ approach and in estimation of pasture DMI [17,31], and were accounted for in pasture DMI calculations in this study.

### 4.2. Dry Matter Intake

Average pasture DMI was only just over a half of total DMI for all farms, despite the grazing-based nature of these systems, and considerably less than the average of 80% of ME intake that occurred on farms 15 years ago [31]. As such, these data support reports that supplementary feeds are increasingly being used by the Australian dairy industry [5,32,33]. However, the range in pasture DMI as a percent of total DMI was larger than that for systems investigated by Powell et al. [21], as the four farms assessed in their study were located in one region, and data were collected only in autumn and spring. Despite different breeds, pasture systems and sampling times in this study the CV for DMI (17%) and range (10 to 29 kg DMI) measured on these farms was low. Mean total DMI for our study was similar to estimates reported by Beever and Doyle [31] of 17 to 18 kg for 600 kg cows grazing pasture on commercial dairy farms. Our data likewise fell within ranges of predicted DMI for cattle at different stages of lactation, that had diverse weights and which received diets with DM digestibilities ranging from 0.5 to 0.8 [34].

Estimates of DMI are known to be influenced by body weight changes that occur in early and late lactation [35]. Losses and gains in body weight cannot be accounted for in short duration on-farm measurements [29], which may have contributed to the observed range in DMI in this study, noting that for only approximately 0.1% of records all or the majority of the cows in the herd were either in late or early lactation. Compounding the difficulty in accounting for liveweight gains and losses is the influence of parity of the cows as well as their production level on the extent of negative energy balance in early lactation [36,37]. Heat and cold stress also affect energy requirements and DMI [38] with lactating dairy cows tolerating temperature ranges from −0.5 °C up to 25 °C [39]. Historical meteorological data give mean minimum and mean maximum temperatures for farm locations in this study of −0.6 and 35 °C, respectively (http://www.bom.gov.au/climate/data/), suggesting that some herds may have experienced temperature stress, although this may not have occurred near dates of farm visits.

Hristov et al. [40] described highly significant influence of DMI on milk yield which we likewise observed between ECM and total DMI for these grazing system herds, as well as weaker but still positive relationships with pasture or supplement DMI. The relationship between ECM and supplement DMI observed for commercial herds in this study was similar to that reported for experimental cows grazing spring pasture and provided with a range of supplements (ECM = 27.96 + 0.30DMI *p* = 0.065; Auldist et al. [25]). Calculated (mean; range) FCEs (1.2; 0.8 to 1.6 kg ECM/kg DMI) for commercial dairy herds in this study were lower than that reported for 13 free stall lactating herds (mean = 1.4, range 1.12 to 1.79 kg 3.5% fat corrected milk/kg DMI) [41] where the higher milk yields and the DMI of the Holsteins fed TMR would have contributed to the differences observed. Grazing herd FCEs indicate lower biological efficiencies for these cows, particularly at the lower end where animals could have been underfed with pasture, or pastures with lower digestibility were provided [31]. In addition, different stages of lactation for the commercial grazing cows would contribute to the range of FCEs calculated. Beever and Doyle [31] reported lower FCEs for later lactation cows grazing paspalum compared with early lactation herds consuming ryegrass pastures. In our study, cows grazed a variety of pasture types ranging from temperate perennial and annual grasses to tropical grass species, legumes, herbs, cereals and brassicas [26]. Despite differences in FCE between US and Australian dairy herds, similarly significant (*p* < 0.001) correlations between FCE and ECM were observed (0.748 [41]; 0.74, this study). However, in contrast to this study, no relationship between FCE and DMI were observed in free stall farms, attributed by Britt et al. [41] to strong seasonal (i.e., temperature) effects on milk production and FCE for US Holstein herds. The relationship between FCE and DMI on grazing system farms was not significant in spring only and that was most likely due to the absence of a relationship with supplement DMI in that season. Thus, in addition to differences in breed, pasture types and seasonal conditions, stage of lactation, parity, dietary CP and weather could have contributed to the small range in DMI estimated (CV < 20%).

In addition to similarities observed between our intake and milk production data and the experimental literature, a relationship between calculated pasture DMI and that estimated by farmers in this study was also observed. Deviations between the calculated DMI and farmer estimates are likely to be due to inaccuracies in calibration of supplementary feeders observed on many of the project farms, which Beever and Doyle [31] consider to be a factor contributing to farmer errors in intake estimations. Moreover, assessments of wastage, required for the ‘Feeding Standards’ approach, are important but are typically not considered in farmer estimates [17,31]. When Heard et al. [17] compared simulated data calculated using the ‘Feeding Standards’ approach with experimentally estimated pasture intake data, a higher (0.76 vs. 0.65 in this study) concordance relationship was observed, and they reported lower correction bias. In commercial settings it will be difficult to estimate precisely the pasture consumed by each animal, but the concordance with farmer estimates observed in our study indicates that, despite limitations of the methodology, the ‘Feeding Standards’ method reflected pasture allocated to commercial herds.

### 4.3. Nutrient Intakes

Dietary P (2.1–7.5 g/kg DMI), Ca (1.7–13.4 g/kg DMI) and Mg (1.4–6.0 g/kg DMI) estimated in this study spanned recommendations for lactating dairy cattle with different levels of milk production [34]. Few incidences of potential dietary deficiencies were observed for P, Ca and Mg, with only a few herds on some farms on some dates having less than recommended nutrient intakes. For P, these dates were always in summer. Dietary K (8.8–39.8 g/kg DMI) was only in excess (>30 g/kg DMI) for a small number of farms on at least one occasion, and for one farm (Farm 1) at all except one occasion. Potassium concentrations of pasture (5.9%), silage (4.5%) and crops (6.5%) provided to these herds would have contributed to high dietary K and could have affected Mg absorption. Fisher et al. [42] described historical increases in pasture and silage K concentrations that are similar to upper concentrations observed in this study. However, despite high K intakes, dietary K balance was never greater than 2.2 [43] and therefore herds were unlikely to be at risk of hypomagnesaemic tetany. In contrast to sporadic high K intakes, elevated dietary S (>4 g/kg DMI) was observed for almost all interview dates for herds on Farms 1, 4 and 19. However, aside from molasses supplement on Farm 4 high pasture and crop S concentrations appeared to contribute to dietary S on the other farms. Compared with TMR diets provided by Idaho farmers [18,19], mean K (1.90%; 1.61 to 2.16%) was similar, and S (0.25%; 0.24 to 0.30%) and Mg (0.37%; 0.34 to 0.41%) were lower than for these grazing system animals (1.87%, 0.31% and 0.46% respectively).

The potential for N and P to be environmental contaminants [44,45] resulted in more studies quantifying intake and excretion of these nutrients on commercial dairy farms compared with K, S, Ca and Mg. For instance, mean dietary P (4.5 g P/kg DM) of grazing herds on these study farms was greater and the ranges generally wider than Spanish (3.98, 2.8 to 5.4 g/kg DMg) [22], Swedish ([3.7, 2.1 to 5.2 g/kg DM) [46], northeast US (4.42, 3.6 to 7.0 g/kg DM) [9], and Wisconsin ([4.0, 2.3 to 8.5 g/kg DM) [47] commercial farms, and well in excess of NRC [35] recommendations of 3.2 to 3.8 g/kg DM for cows producing 22 to 55 kg milk/day. Mean P concentration of dietary components (0.47%, 0.01 to 3.99%) provided to the cows in this study was less than for Swedish rations (0.6%, 0.25 to 1.1%) [46], and Wisconsin (1..8%, 0.24 to 3.58%) [47]), and closer to that of Idaho (0.49%, 0.41 to 0.50%) [19]) farms. Arriaga et al. [22] attributed high P intakes in their study mainly to grass silage, although concentrates had higher P concentrations. In our study, as reported elsewhere [9,47], mineral supplements and concentrates contributed the highest P concentrations to diets. Despite these reported differences, daily P intakes on Spanish and Wisconsin commercial farms of approximately 84 g [22,47] were close to that estimated for grazing system farms in our study where 72% of records were above dietary P recommendations. When P intake was averaged for each farm in this study, approximately 81% of farms fed above recommended P thresholds for high producing cows, slightly less than that reported for Wisconsin farmers [47], although farms in our study did not always overfeed P, and milk production was often lower than the basis of the recommendations.

Mean dietary CP concentration (19%) for cows in this study was slightly higher than both Australian (16%) [34] and NRC [18%, 35] recommendations, and greater than observed on Idaho (17.6%) [19], Wisconsin (17.2%) [20] and Spanish (16.4%) [22] farms. However, mean daily N intake for Australian animals was only slightly less than that reported for Spanish cows. Consequently, most farms in our study appear to be overfeeding dietary protein with only 12% of the data lower than the recommended threshold of 400 g N/day [48,49]. Similarly, Jonker et al. (2002) reported that 71% of Chesapeake Bay farmers overfed N in their survey. Thus, at the upper end of CP intake, considerable excretion of N would be expected in these systems.

### 4.4. Nutrient Excretion and Animal Use Efficiency

Mean N and P excretion (g/cow per day) calculated for these grazing system farms fell within ranges collated from a review of experimental literature [50]. When results from this study for K, S, Ca and Mg excretion were compared with the few published data for commercial herds fed TMR in California [8,51], K was between 14 and 44 g/cow per day greater than observed for the TMR herds, and at the upper end of experimental data collated for the review mentioned above. Greater median, 25th and 75th quartile (2.0%, 1.67%, 2.5%) dietary K for grazing cows, compared to those for the California animals (1.62%, 1.46%, 1.75%, respectively) [8], indicates possible greater excretion by grazing herds. Our observed K excretion contrasts with Ca, P, and Mg where the California herds excreted more on a daily basis (58, from 8 to 13, between 15 and 23 g/cow per day, respectively). The greater mineral excretion by the California herds can be due to the 30% higher milk yields of those animals and the associated greater dietary DM intake to support higher body weight. Sulphur excretion by the TMR herds varied when compared with this grazing study, with less excreted in one report and more in the other. Ranges (maximum minus minimum) in excretion were greater for Australian grazing systems than international N (410 g/cow per day) and K (143 g/cow per day) data [50], but were similar for P (107 g/cow per day). Likewise, for commercial TMR herds [8,51] ranges for K and S excreted were approximately half that of the grazing cows, but were comparable for P, Ca and Mg.

Management factors can influence nutrient excretion, and as observed by Borsting et al. [52], N excretion by grazing lactating cows increased as milk yield. Arriaga et al. [22] describe ‘intensification’ management factors such as herd size and land availability that influenced N more than P excretion. On the grazing system farms stocking rate, herd size and annual milk yield strongly influenced N excretion, whereas P, K and Mg excretion were significantly affected by the feed ME requirements brought onto farms. The contribution of dietary minerals and concentrates to P and Mg intake and pasture to K intake could explain the relationships observed.

Animal nutrient use efficiencies, based on dietary nutrients and nutrients secreted in milk, are often considered a useful means of comparing the potential for environmental impact. Estimating animal use efficiencies and excretion of macrominerals is both important for animal health and productivity as well as for minimizing salt loads to soils [53]. On commercial farms animal N use efficiencies ranged from 18% to 33% [20], from 19.2% to 32.3% [22], and from 24.5% to 32.3% [54], while feed P use efficiencies were between 19.3% and 44.7% [22] and between 18% and 35% [20]. Mean N and P use efficiencies for lactating cows on our grazing system farms were lower than reported in the literature, with wider ranges in N and P use and lower minimum efficiencies. Limited data are available in the literature for K, S, Mg and Ca feed use efficiencies on commercial farms. Using excretion and milk nutrient data for TMR-fed herds in California [8], animal feed use efficiencies were calculated that were equivalent (S, Mg) or less than (Ca) those for the grazing systems in this study.

### 4.5. Estimating Nutrient Excretion for Grazing System Farms

This paper addresses the requirement for technological solutions to improve nutrient management [55], and is particularly relevant for grazing systems globally. Nutrient management programs for Australian grazing systems are largely based on improving fertilizer management, focusing on the assessment of soil test values and quantification of input output farm-gate budgets [56]). As a consequence, these programs do not account for the large within-farm flow of nutrients through dairy cows that is expected in these systems and the variety of places where these animals are likely to excrete [50]. On many grazing system farms animals were spending less time in grazed paddocks and more time in paddocks closest to dairy sheds were soil test levels were elevated [50,57].

The methodology to estimate dietary intake of grazing cows described in this paper can be used for calculating nutrient excretion and to develop within-farm nutrient budgets. The herd data (milk production, supplement DMI, stage of lactation etc.) required for the daily ‘Feeding Standards’ approach are readily available on commercial dairy farms. Dates of data collection should represent major seasonal influences on diet and milk production, which for Australian grazing systems would be at least once in each season. However, as farmers seek to increase productivity, individualised feeding systems are recommended [52,58] and the associated automation of these systems should be able to provide the daily cow specific diet, lactation and even liveweight or body condition score information that will improve estimation of pasture DMI.

This approach for calculating excreted nutrients can be used to identify nutrient management points of intervention on grazing system dairy farms. By combining excretion data with information about the locations within farms where animals are held [50], deposited nutrient loads can be calculated which will indicate nutrient accumulation zones where the potential risk of nutrient loss is high. Estimating nutrient deposition by cows should improve manure and effluent management as a number of surveys have shown that farmers often did not know manure N applied [20] or rarely tested their manure before application [59], indicating a need for methods to more reliably estimate manure nutrient application, particularly for grazing system farms.

## 5. Conclusions

This work demonstrated that the daily pasture DMI of lactating herds can be estimated with a modified energy requirement (‘Feeding Standards’) approach using data readily available on commercial dairy farms. The calculated intakes, which are comparable to those reported in the literature and are concordant with farmer estimates of feed provided, indicate the feasibility of this approach for estimating nutrients excreted by lactating cows in grazing systems. Using nutrient analyses of all feeds offered on farms instead of book values, including estimates of feed wastage, and ensuring accurate calibration of supplementary feeders are likely to have contributed to the agreement of calculated pasture DMI with farmer estimates of pasture consumed. More accurate liveweight data could improve DMI estimates, with sensitivity analysis indicating a less than 5% difference in calculated values. Using this approach for lactating herds grazing on commercial dairy farms, we showed that only approximately half the animals’ total DMI consisted of pasture, and calculated herd N and P intakes were greater than recommended in the literature. Daily excreted N, P, K, S, Ca and Mg were calculated for these grazing herds, with low feed N and P use efficiencies indicating the potential for nutrient losses that will need to be managed on commercial farms.

## Figures and Tables

**Table 1:** Summary statistics for characteristics of milking herds and the 43 grazing system dairy farms collected at the five interview dates.

Herd and Farm Characteristics	Minimum	Mean	Median	Maximum	SD	CV
Herd size ^1^	30	267	212	1350	202.2	76%
Number of lactating cows ^2^						
Primiparous	0	66	55	230	46.5	71%
Multiparous	10	201	153	1330	178.3	89%
Early	0	68	35	850	114.0	169%
Mid	0	119	85	900	132.2	111%
Late	0	80	45	800	110.0	137%
Average age (y)	2.7	4.0	4.0	4.5	0.2	6%
Liveweight ^3^ (kg)	430	525	500	680	41.4	8%
Milk						
Yield ^4^ (L/day)	7	21	22	36	5.7	27%
ECM ^5^ (kg/day)	9	22	22	36	5.3	24%
Protein (g/L)	30.9	33.1	33.0	38.3	1.57	5%
Fat (g/L)	36.5	40.9	40.2	51.3	2.97	7%
Distance walked (km)	0.00	0.99	0.84	4.08	0.662	67%

^1^ The number of cows in each herd on each farm at each visit, noting that on some visits to some farms, more than one herd were present while other farms always had at least two herds. On most farms, the calving pattern was seasonal or split, rather than year-round. ^2^ Number of lactating cows that are primiparous, multiparous, in early (≤102 days), mid (>102, ≤ 204 days), late (>204, ≤ 305 days) lactation. ^3^ Liveweight estimated by the farmer. ^4^ Average milk volume produced per cow given by farmer. ^5^ ECM, energy corrected milk calculated as (milk yield (L) × 1.0295 × (376 × fat (%) + 209 × protein (%) + 948))/3138) [25].

**Table 2:** Summary statistics of milk volume produced by the herd, and calculated per lactating cow and per heifer, the calculated herd metabolizable energy (ME) required for maintenance, grazing, pregnancy, milk production, activity, and total ME per cow for lactating cows at five occasions on 43 farms (*n* = 227).

Summary Statistics	Milk Produced			Metabolizable Energy (MJ/Herd Per Day)					Total ME
	L ^1^/herd	L ^2^/cow	L ^3^/heifer	Maintenance	Grazing	Pregnancy	Milk Production	Activity	(MJ/Cow Per Day)
Minimum	438	7	0	1571	157	0	2878	238	116
Mean	5937	23	18	14,590	1459	460	34,249	2646	195
Median	4218	23	18	11,473	1147	283	24,798	1595	194
Maximum	37,800	38	31	80,150	8015	4140	208,050	20,186	289
SD	5425.7	6.0	5.3	11,180.6	1118.1	522.8	30,284.9	2826.4	33.4
Var	29,438,659.6	36.3	27.7	125,006,498.3	1,250,065.0	273,271.8	917,172,365.3	7,988,323.0	1115.5
CV	91%	27%	29%	77%	77%	114%	88%	107%	17%
Skew	3.0	0.1	−0.5	2.4	2.4	3.3	2.9	2.8	0.3
Kurt	13.1	0.0	1.4	8.5	8.5	15.3	11.9	10.4	0.2

^1^ Calculated from the average per cow milk produced (Table 1) and the number of cows in the herd. ^2^ Calculated for the number of multiparous cows in the herd. ^3^ Calculated for the primiparous cows in the herd.

**Table 3:** Mean nutrient (minimum-maximum) concentrations in samples of feeds (pasture and supplements) consumed by grazing herds on five occasions on 43 dairy farms.

Feed Types ^1^	Number	ME (MJ/kg DM)	N (%)	P (%)	K (%)	S (%)	Ca (%)	Mg (%)
Pasture	248	11 (7–13)	3.7 (1.0–5.8)	0.45 (0.11–0.95)	2.8 (0.5–5.9)	0.34 (0.12–0.60)	0.59 (0.11–2.21)	0.31 (0.11–2.28)
Concentrates	163	13 (12–15)	2.5 (1.0–5.2)	0.57 (0.14–1.99)	0.6 (0.3–1.5)	0.21 (0.10–0.46)	1.15 (0.01–4.92)	0.45 (0.03–2.35)
Silage	114	9 (7–11)	2.4 (1.1–4.4)	0.39 (0.13–0.76)	2.5 (0.7–4.5)	0.26 (0.06–0.55)	0.52 (0.12–1.09)	0.25 (0.13–0.63)
Hay	94	8 (4–11)	1.8 (0.2–4.4)	0.25 (0.01–0.66)	1.7 (0.7–2.7)	0.19 (0.04–0.64)	0.48 (0.10–1.99)	0.21 (0.07–0.87)
By-products ^2^	76	12 (0–17)	3.3 (0.1–8.5)	0.53 (0.01–1.45)	2 (0–5)	0.47 (0.02–1.33)	0.54 (0.01–1.17)	0.34 (0–0.74)
Crops	23	10 (8–11)	2.6 (1.4–3.9)	0.32 (0.2–0.55)	3.0 (1.4–6.5)	0.49 (0.09–1.05)	1.02 (0.30–1.85)	0.50 (0.26–0.98)
Mixed grain	33	13 (11–16)	2.6 (1.4–3.5)	0.40 (0.22–0.59)	0.6 (0.4–0.8)	0.19 (0.10–0.29)	0.24 (0.04–0.90)	0.23 (0.11–0.56)
Grain	32	13 (12–15)	2.5 (1.7–6.5)	0.36 (0.22–1.19)	0.6 (0.3–3.4)	0.17 (0.09–0.73)	0.11 (0.01–0.56)	0.15 (0.10–0.60)
Mineral ^3^	30	9 (2–13)	0.4 (0.0–1.7)	1.81 (0–3.99)	0.6 (0–1.9)	1.07 (0–2.55)	10 (0.01–40)	3.63 (0–8.01)
TMR ^4^	12	9 (9–10)	2.7 (1.8–3.5)	0.45 (0.28–0.55)	1.9 (1.4–2.3)	0.25 (0.18–0.30)	0.90 (0.43–1.23)	0.28 (0.25–0.31)
Organic ^5^	4	13	0.5	0.14	1.3	1.12	11.97	7.11

^1^ The range of feeds provided to lactating herds on farms at all five visits, where all feed types except pasture were considered to be supplementary. More details can be found in Rugoho, et al. [26]. ^2^ By-products include a variety of meals (soybean, canola, cottonseed, linseed), pea pollard, palm kernels, bread, citrus pulp, molasses, brewer’s grain, apple cider vinegar, tomatoes, almond hulls, raw sugar and cod liver oil, fed at various interview dates on 16 farms. ^3^ In addition to several proprietary commercial mineral products, this feed type also included sea salt, bicarb, limestone and urea fed on eight farms. ^4^ TMR—total mixed rations fed predominantly on one farm, but also included hay and silage based feed on two other farms. ^5^ The organic feed type was a kelp/dolomite mix fed on one farm at the first four interviews.

**Table 4:** Summary statistics for supplement dry matter (DM) and metabolizable energy (ME) fed and calculated intakes of ME, DM and nutrients from pasture of lactating herds on five occasions on 43 farms (*n* = 227).

Summary Statistics	Supplement Fed ^1^		Calculated Intake from Pasture							
	DM	ME	ME ^2^	DM	N	P	K	S	Ca	Mg
	kg/Cow Per Day	MJ/Cow Per Day	MJ/Cow Per Day	kg/Cow Per Day	g/Cow Per Day					
Minimum	1.1	13	0.4	0.04	1.2	0.2	0.9	0.2	0.4	0.1
Mean	9.2	104	98	9.1	336	42	260	32	55	28
Median	8.6	97	97	9.1	320	39	247	30	52	25
Maximum	25.4	251	236	22.5	787	117	686	81	190	267
SD	4.91	52.2	45.4	4.7	165.9	23.4	141.4	17.6	33.3	21.4
Var	24.07	2725.0	2056.7	21.9	27,531.5	546.6	19,988.9	309.3	1110.3	459.8
CV	54%	50%	47%	51%	49%	56%	54%	54%	61%	76%
Skew	0.56	0.5	0.1	0.1	0.2	0.7	0.4	0.5	0.8	6.6
Kurt	0.08	−0.1	−0.2	−0.2	−0.4	0.4	−0.1	−0.1	0.8	71.4

^1^ Total supplements fed, accounting for wastage (as estimated by the farmer) and reflecting actual amounts provided by correcting inaccurately calibrated dairy shed feeders. ^2^ Pasture ME calculated by subtracting supplement ME from total ME requirements.

**Table 5:** Linear regression relationships and *p* value for relationships between per cow energy corrected milk (ECM) or feed conversion efficiency (FCE) and total dry matter intake (DMI) or supplement DMI (DMI_SUP_) for each interview and for all data. The range in adjusted R^2^ for all relationships is provided.

Time Periods ^1^	ECM ^2^		ECM ^3^		FCE ^4^	
	(kg/day)	*p*	(kg/day)	*p*	(kg ECM/kg DMI)	*p*
Summer						
January/February 2008	1.55 DMI ^2^−7.36	2.87 × 10^−14^	0.39 DMI_sup_^3^ + 16.99	0.00482	0.023 DMI + 0.72	0.00715
January/February 2009	1.51 DMI−5.39	<2 × 10^−16^	0.59 DMI_sup_ + 15.53	1.56 × 10^−6^	0.015 DMI + 0.93	0.0217
Autumn						
May 2008	1.89 DMI−10.19	<2e × 10^−16^	0.63 DMI_sup_ + 14.65	0.00148	0.042 DMI + 0.56	2.64 × 10^−7^
Winter						
July/August 2008	1.58 DMI−4.08	<2e × 10^−16^	0.72 DMI_sup_ + 17.71	0.000126	0.016 DMI + 1.07	0.024
Spring						
October/November 2008	1.33 DMI−1.34	1.67 × 10^−15^	0.18 DMI_sup_ + 24.15	0.238	0.007 DMI + 1.12	0.25
All data	1.51 DMI−4.89	<2 × 10^−16^	0.39 DMI_sup_ + 18.94	7.81 × 10^−8^	0.018 DMI + 0.92	2.65 × 10^−7^
Adjusted R^2^	0.72 < R^2^ > 0.86		0.14 < R^2^ > 0.40		0.09 < R^2^ > 0.45	

^1^ Time periods used in linear regression analysis were each interview data (categorised according to season) and combined interview dates (all data). ^2,4^ Regression relationship between ECM or FCE and total DMI. ^3^ Regression relationship between ECM and supplement DMI (DMI_SUP_). Linear regression was performed using R Studio © 0.99.891 (R version 3.2.3).

**Table 6:** Pearson correlation coefficient and statistical significance (*F-prob*) for correlations between milk production and intake parameters for lactating dairy herds on 43 grazing system dairy farms.

Correlation Variables		R	*F-prob*
ECM	DMI	0.87	0.000
ECM	FCE	0.75	0.000
ECM	DMIpas	0.20	0.002
ECM	DMIsup	0.36	1.69 × 10^−8^
ECM	Milkfat	−0.51	3.33 × 10^−16^
ECM	Milkprot	−0.19	0.003
FCE	DMI	0.33	3.44 × 10^−7^
FCE	DMIpas	0.006	ns
FCE	DMIsup	0.21	0.002
FCE	Milkfat	−0.31	2.01 × 10^−6^
FCE	Milkprot	−0.05	ns
DMI	DMIpas	0.29	1.28 × 10^−5^
DMI	DMIsup	0.37	9.89 × 10^−9^
DMI	Milkfat	−0.52	0.000
DMI	Milkprot	−0.28	1.34 × 10^−5^
DMIpas	DMIsup	−0.79	0.000
DMIpas	Milkfat	−0.22	0.0007
DMIpas	Milkprot	−0.12	0.067
DMIsup	Milkfat	−0.12	0.068
DMIsup	Milkprot	−0.07	ns
Milkfat	Milkprot	0.63	0.000

ECM, energy corrected milk [27]; DMI, estimated total dry matter intake; FCE, feed conversion efficiency (kg ECM/kg DMI); DMIpas, estimated pasture dry matter intake; DMIsup, supplement dry matter fed (accounting for wastage); Milkfat, milk fat (%) measured by chemical analysis; Milkprot, milk protein (%) measured by chemical analysis.

**Table 7:** Summary statistics for calculated nutrient intake (g/cow per day), excretion (g/cow per day) and animal nutrient use efficiencies (%) across 43 grazing system dairy farms for five interviews.

Summary Statistics	N	P	K	S	Ca	Mg
**Nutrient Intake (g/Cow Per Day)**						
Minimum	268	27	175	24	30	22
Mean	545	81	372	52	116	54
Median	543	81	364	51	111	52
Maximum	983	155	703	108	236	101
SD	129.4	23.8	105.2	13.8	41.8	15.0
Var	16,755.4	568.5	11,058.5	190.1	1744.4	225.1
CV	24%	29%	28%	26%	36%	28%
**Nutrient Excretion (g/Cow Per Day)**						
Minimum	199	20	140	19	10	21
Mean	433	61	341	44	92	52
Median	429	60	335	42	89	50
Maximum	793	132	671	101	210	98
SD	110.3	20.7	102.7	13.0	40.6	14.7
Var	12,171.6	429.7	10,537.9	169.1	1651.5	216.0
CV	25%	34%	30%	30%	44%	28%
**Animal Nutrient Use Efficiency (%)**						
Minimum	11	4	2	3	8	2
Mean	21	25	9	16	23	4
Median	21	24	8	15	21	4
Maximum	39	48	21	48	76	8
SD	4.3	6.6	3.3	7.1	10.8	1.2
Var	18.1	43.1	11.0	51.1	116.2	1.5
CV	20%	26%	38%	44%	46%	28%

**Table 8:** *F-prob* (effects) estimates for residual maximum likelihood (REML) analysis of daily nutrient excretion and characteristics of these grazing system dairy farms.

Farm Characteristics	Daily Excretion (g/Cow Per Day)					
	N	P	K	S	Ca	Mg
Farm area ^1^ (ha)	0.013 (0.1557)	ns	ns	ns	ns	ns
Herd size ^2^	<0.001 (0.1238)	ns	ns	0.001 (0.01197)	0.027 (−0.02639)	ns
Stocking rate ^3^ (cows/ha)	0.004 (25.12)	ns	ns	<0.001 (3.582)	0.046 (−6.511)	ns
Total milk produced ^4^ (L)	<0.001 (1.746 × 10^−5^)	0.055	ns	0.001 (1.332 × 10^−6^)	ns	ns
Per cow milk produced ^5^ (L/cow)	<0.001 (0.02555)	<0.001 (0.004571)	ns	0.089 (0.0009186)	ns	<0.001 (0.003544)
Per ha milk produced ^6^ (L/ha)	<0.001 (0.004041)	0.019 (0.0004390)	ns	<0.001 (0.0003972)	ns	ns
Percent of farmland irrigated	ns ^8^	0.057 (−0.08398)	ns	ns	ns	ns
Feed ME ^7^ (%)	ns	<0.001 (0.4266)	0.007 (−1.312)	ns	ns	<0.001 (0.2439)

^1^ Total land area visited by lactating cows, which includes grazed paddocks, cropping, feeding area such as feedpads and sacrifice paddocks, laneways, the dairy shed and associated yards. ^2^ Total number of lactating and dry cows, and heifers about to calve for the first time averaged from data collected at 5 quarterly on farm visits. ^3^ Cow numbers divided by the farm area. ^4^ Total liters of milk produced by the farm. ^5^ Calculated by dividing total production by the herd size for each farm. ^6^ Calculated by dividing total production by the farm area for each farm. ^7^ Feed metabolizable energy requirements (ME) as a percentage of total ME requirements that is imported on to farms. ^8^ *F prob* > 0.100.

## Supplementary Materials

The following are available online at https://www.mdpi.com/2076-2615/10/3/390/s1. Figure S1: Map showing the location of project farms across temperate, arid, subtropical and tropical zones of Australia. Figure S2: Relationship between calculated pasture dry matter intakes and farmer estimates of what was provided to their lactating herds. Table S1: Herd, milk production, fat and protein concentration and terrain data, as well as calculated milk nutrients, supplement, pasture and total DMI, and nutrient intake, excretion and use efficiency for lactating cows at 5 visits over 4 seasons on 43 commercial dairy farms. Table S2: Amounts and nutrient contents of all feed types consumed by the lactating herds on 5 different visits to 43 dairy farms; including wastage, estimates by farmer of pasture and where feeder weights were adjusted.
