# Supplementary material for: Nutrient Intake, Excretion and Use Efficiency of Grazing Lactating Herds on Commercial Dairy Farms
Source: Animals (Basel). 2020 Feb 28;10(3):390. doi: 10.3390/ani10030390 (PMC7143236; doi:10.3390/ani10030390)
Supplement: Supplementary file 1 [file animals-10-00390-s001.zip › animals-723453-supplementary/animals-723453-supplementary v2.docx]

Nutrient intake, excretion and use efficiency of grazing lactating herds on commercial dairy farms

S. R. Aarons, C. J. P. Gourley and J. M. Powell


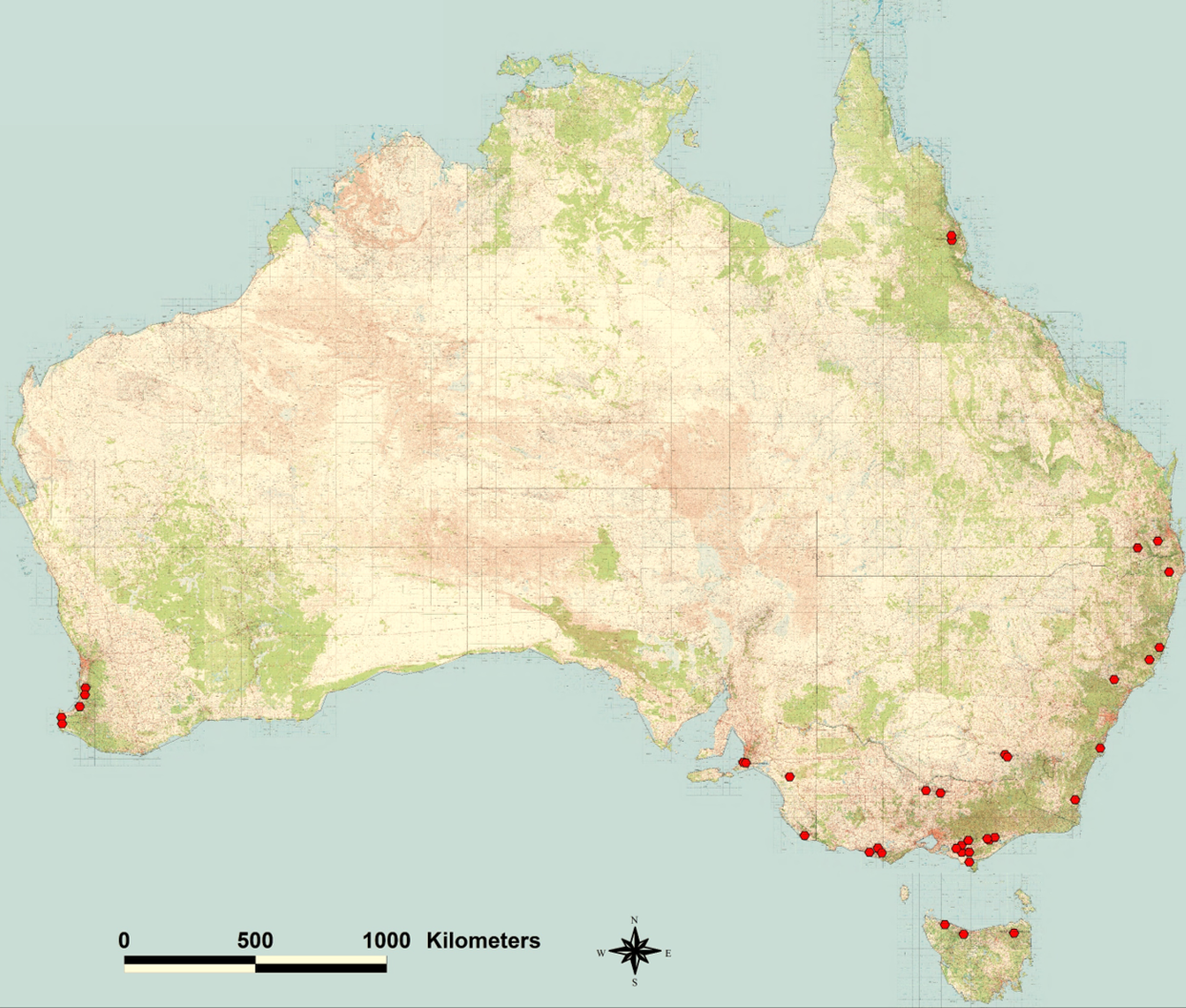


Figure S1. Map showing the location of the 43 study farms located across temperate, arid, sub-tropical and tropical zones of Australia.

Figure S2. Lin’s Concordance relationship between calculated pasture dry matter intakes and farmer estimates of what was provided to their lactating herds.
