# Supplementary material for: Nutrient Intake, Excretion and Use Efficiency of Grazing Lactating Herds on Commercial Dairy Farms
Source: Animals (Basel). 2020 Feb 28;10(3):390. doi: 10.3390/ani10030390 (PMC7143236; doi:10.3390/ani10030390)
Supplement: Supplementary file 1 [file animals-10-00390-s001.zip › Nutrient intake excrn_Suppl Feb2020.docx]

Nutrient intake, excretion and use efficiency of grazing lactating herds on commercial dairy farms

S. R. Aarons, C. J. P. Gourley, and J. M. Powell

**Supplementary material**


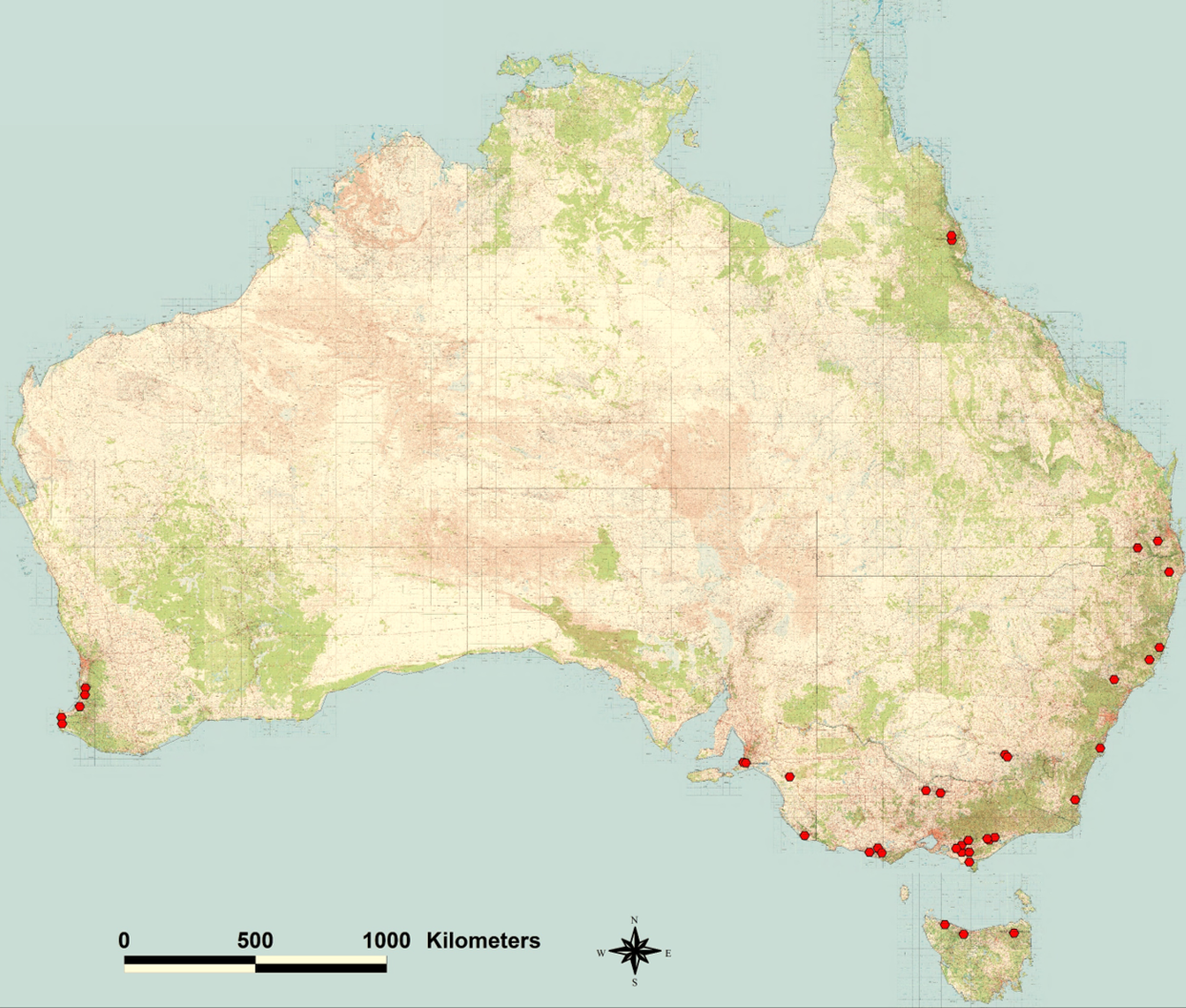


Supplementary Figure 1 Map showing the location of the 43 study farms located across temperate, arid, sub-tropical and tropical zones of Australia.

Supplementary Figure 2 Lin’s Concordance relationship between calculated pasture dry matter intakes and farmer estimates of what was provided to their lactating herds.
